# Supplementary material for: Education Intervention Has the Potential to Improve Short-Term Dietary Pattern among Older Adults with Undernutrition
Source: Geriatrics (Basel). 2023 May 17;8(3):56. doi: 10.3390/geriatrics8030056 (PMC10204358; doi:10.3390/geriatrics8030056)
Supplement: Supplementary file 1 [file geriatrics-08-00056-s001.zip › geriatrics-2267043-supplementary.pdf]

Supplementary materials

S1-Supplementary Table - criteria used to describe an older adult as undernourished (overall)

**Criteria**	**Cut-off value for undernutrition in men**	**Cut-off value for undernutrition in women**
BMI	< 18.5 $Kg.M^2$	< 18.5 $Kg.M^2$
MUAC	≤ 23.0 cm	≤ 22.0 cm
Body fat percentage	< 14.0 %	< 26.0%
Body muscle percentage	< 34.4%	< 26.6%

S2-Tool to assess the validity of the intervention

		Not Relevant	Somewhat relevant	Quite Relevant	Highly Relevant
1	The activities are relevant to the study's objectives.				
2	The activities appropriately cover the study's objectives.				
3	The number of activities is sufficient.				
4	The activities are easy to understand for the target group.				
5	The activities do not need any specific background.				
6	The activities have enough and understandable details.				
7	The details of activities clearly represent the concepts.				
8	The module has logical arrangement and sequence of improving dietary pattern.				
9	The module has accurate content.				
10	The module can be used as a nutrition education package.				
11	The activities are adopted efficiently with the local culture.				
12	The activities are clearly written.				
13	The activities are suitable for older people				
14	The activities are executable with minimum facilities and equipment.				
15	Overall- the module has a clear content.				
16	Overall- the module is developed sufficiently regarding to the study's objectives and target group.				

S3-List of activities conducted in the intervention

Activity 1

Identifying food items belong to each of the 6 food groups

- Be seated in 6 circles.
- Provide pictures of various food items to each group.
- Explain and advise them to sort the pictures in to 6 food categories as mentioned below.

  Starchy food

  Proteins

  Legumes/ Lentils

  Fruits

  Vegetables

  Dairy products

- Give the help of the facilitator accordingly.
- Appraise the groups who has completed the activity successfully.
- Discussion points by the facilitator.

Activity 2

Identifying low cost high nutritious food items

- Be seated in 6 circles.
- Produce each group the following food items

  An egg

  Some sprats

  Slice of bread

  Wedge of cheese

Cup of yoghurt

A potato

Coconut roti

- Ask the participants to choose low-cost but highly nutritious food items out of the above groups.
- Facilitator's discussion on learning points.
- Appraise the participants for coming up with correct answers.

Activity 3

Myths and misconceptions related to diet

A facilitator is coming up with common myths and misconceptions related to diet in the community like hot and cold food items, food items bad for flem and diet during illnesses.

Ask the participants about the myths they are having.

Gradually convince them about the bad effect of myths on nutrition.

Expand the discussion focusing in to individual issues and clarifications related to myths and misconceptions.

Activity 4

Importance of home gardening

Invite a selected older adult from the same community area who is maintaining a home garden to the intervention session.

Ask her to share their experiences with the participants.

The facilitator is directing the discussion.

Discuss the alternative methods of doing home gardening within the limited space available.

Inform regarding supporting resources such as agricultural officer availability

Activity 5

Concept of Healthy plate

Be seated in 6 circles.

Provide all the participants with a paper plate and a colored pencil.

Advise them to listen to the facilitator carefully and follow the instructions.

Ask the participants to mark the sizes of various food groups (starchy food, fruits and vegetables, proteins, legumes and lentils and milk products) in the paper plate according to their knowledge and show you.

Correct them by giving the recommended portion sizes of food groups.

Appraise the older adults who has done the activity correctly.

Activity 6

Getting participation of older adults in the decision-making process related to the diet.

Be seated in 6 circles with the caregiver if available.

Invite a volunteer caregiver to come to the front and share an instance where he/she has got involved with the older adult in the decision-making process related to the diet.

Like that share the experiences of some volunteer caregivers.

Same time, the facilitator is trying to convince caregivers about the importance of getting older adults participation into the decision-making process related to the diet.

Invite a few elders to the front and get their views on this issue. Whether they to participate in to the decision-making related to diet like selecting food items to meals or preparing meals.

S4-Feedback Form

Serial no………………

Answer each question by marking your response in the relevant cages.

1. The content included in the intervention was
   Satisfactory ☐
   Unsatisfactory ☐

2. The way of presentation was
   Clear ☐
   Unclear ☐

3. Mode of delivery of the lessons were
   Satisfactory ☐
   Unsatisfactory ☐

4. Overall rating of the intervention
   Satisfactory ☐
   Unsatisfactory ☐

5. Suggestions for improvement

   ……………………………………………………………..

S5

Confidential

Undernutrition, dietary pattern, associated factors and effectiveness of a nutrition education intervention in improving undernutrition among elderly in Colombo District

Questionnaire

DS Division: Serial no.

GN Division:

Name of the elderly:

Address: Date:

Part one - Assessment of factors associated with undernutrition

Part A- Socio-demographic factors

1. Date of birth Day……… Month……. year…………
Age in years ………………………

2. Sex

1. Male	
2.Female	

3. Ethnicity

1.Sinhala	
2.Tamil	
3.Muslim	
4.Burger	
5.Other	

4. Religion

1.Buddhist	
2.Christian	
3.Hindu	
4.Islam	
5.other	

5. Marital status

1.Married	
2.Widowed	
3.Divorced	
4.Unmarried	
5.Separated	
6.Living together	

6. What is your living environment?

1.Urban - Residential	
2.Urban – Slum	
3.Rural	
4.Estate	

7. What is your highest educational achievement?

1.No schooling	
2.Grade 1-5	
3.Grade 6-10	
4.G.C.E O/L passed	
5. G.C.E. A/L passed	
6.Degree and Postgraduate	

8. What is your employment status?

1.Currently employed	
2.Retired	
3.Never employed	

9. What is your monthly income in rupees?

1.No income	
2.< 5000	
3.5000 – 10000	
4.10,001 – 15, 000	
5.>15, 000	

10. What is your Source of income? (can mark more than one)

1.Employment / pension	
2.Assets / Bank accounts	
3.House rentals	
3.Family members support	
4.Relatives support	

5.Government assistance	

11. How many others living with you & the relationship with you? Total…….

Relationship
1.
2.
3.
4.
5.
6.
7.
8.
9.
10.

12. Who is your primary care giver?

1.husband/ wife	
2.Son/Daughter	
3.Son in law/Daughter in law	
4.Siblings	
5.Grand children	
6.Relative	
7. Other	

13. Do you look after your grandchildren?

1.Yes	
2.No	

14. What is your level of satisfaction on family support?

1.Very satisfied	
2.Satisfied	
3.Dissatisfied	

Part B- Factors associated with health status and disabilities

15. Are you suffering from following chronic medical conditions?

1.Diabetes Mellitus	
2.Hypertension	
3.Heart diseases	
4.Renal failure	
5.Asthma/COPD	

6.Convulsions	
7.Any other	

16.Were you hospitalized during the past 3 months?

1.Yes	
2.No	

16. If yes, duration of hospitalization………………

18. Where do you go to seek treatment? (Mark the most frequently visited place)

1.Government – western Institution	
2.Government – indigenous Institution	
3.Private – western institution	
4.Private – indigenous institution	
5.Spiritual/ religious activities	
6.Other	

19. How many drugs do you use for co- morbidities per day?.................

20. Are you having food allergies?

1.Yes	
2.No	

21. Are you having disabilities?

1.Yes	
2.No	

22. What are the physical disabilities you are having and mark whether you are using disability aids?

Physical disability	Present or not
1.Hearing	
2.Smell	
3.Taste	

4.vision	
5.Chewing	
6.Musculoskeletal problems	
7.Any other	

23. What are the Psychological disabilities you are having and mark whether you are taking treatment?

Psychological disability	Present or not
1.Depression	
2.Schizophinia	
3.Any other	

<u>Part C – Factors associated with behaviors</u>

24. Do you currently smoke?

1.Yes	
2.No	

25. Are you currently using alcohol?

1.Yes	
2.No	

26. If yes, what is the type and amount (per week) of alcohol you are consuming?

Type of alcohol		Amount
1.Arrack		
2.Beer		
3.Kasippu		
4.Other		

27. Are you chewing betel?

Yes	
No	

28. If yes, what is the frequency per day?.................

Part Two – Assessment of factors associated with dietary pattern

29. How much of responsibility do you have for the following?

	Little or none	Average	Most or all
1.Food shopping			
2.Planning meals			
3.Preparing meals			

30. In a typical week, where are your meals prepared mostly?

	At home	Out	Do not eat meals
1.Breakfast			
2.Lunch			
3.Dinner			

31. Do you skip meals?

1.Yes	
2.no	

32. How do you receive nutritional advises? (Can give more than one response)

1.Not receiving	
2.General practitioner	
3.Hospital	
4.Public Health Midwife	
5.Others specify….	

33. Availability of a home garden?

1.Yes	
2.No	

Part Three – Assessment of the dietary pattern

24 hour dietary recall sheet

**MEAL**	**Items consumed**	**Amount**
**Break fast**		

**Morning snack**		
**Lunch**		
**Afternoon snack**		
**Dinner**		
**Other**		

<u>Part Four- Assessment of nutritional status</u>

<u>Anthropometric measurements and Body composition measurements</u>

	First time	second time	Average
**Weight**			
**Height**			
**Arm span length**			
**Mid upper arm circumference**			
**Body fat percentage**			
**Body muscle percentage**			

S6- showing the different food groups, recommended daily servings and assigned scores

Food group	Recommended daily serving	Assigned score out of 20
1. Cereal, roots, or equivalents (starchy food)	4	4
2.vegetables	2	4
3. Fruits	2	4
4. Meat, fish, eggs or alteration	1	2
5. Legumes / Lentils	1	2
6. Milk/ Dairy products	2	4

*One serving is calculated according to the calculations mentioned in food based dietary guidelines in Sri Lanka.

S7- showing the serving sizes of different food groups

Food group	Serving size
**Cereal and starchy foods**	
Cooked rice / milk rice/pasta /noodles	1 cup (130 – 140g)
Bread	1 slice (50 g)
String hoppers	6
Hoppers /dosai	2
Roti	1
Pittu	1piece
**Vegetables**	
Cooked vegetables (fruit & leafy vegetables)	3 tbsp (1/2 cup)
Raw salads	1 cup (200 ml)
**Fruits**	
Medium size fruit	1 (1 Banana/ 1 Orange)
Cut fruit / fruit salad	½ cup
Dried fruit	2 tbsp (20 -30 g)

**Fish, Pulses, Dried fish, Eggs, poultry & meat**	
Cooked fish/ poultry / meat	30 g
Cooked pulses	3 tbsp
Eggs	1
Dried fish	15 g
**Milk & Dairy products**	
Milk	1 cup (200 ml)
Youghurt/ curd	1 cup (100 ml)
Milk powder	30 g (2 tbsp)
**Nuts and Oil seeds**	1 tbsp (15 g)

*1 cup = 200 ml tea cup

**Tbsp = table spoon (15 g)

***Coconut shell spoon= 100 g

****Cooked items quantities measured without gravy.
